# Open Pilonidal Excision as a Translational Human Model for Wound Healing and Skin Regeneration Research

**DOI:** 10.3390/biomedicines14040751

**Published:** 2026-03-26

**Authors:** Dimitrios Vardakostas, Zoe Garoufalia, Anastassios Philippou, Dimitrios Mantas

**Affiliations:** 1 Renal Transplant Unit, “Laiko” General Hospital, 11527 Athens, Greece; 2 “Hygeia” Hospital, 15123 Athens, Greece; zgaroufalia@hygeia.gr; 3 Department of Physiology, Medical School, National and Kapodistrian University of Athens, 11527 Athens, Greece; tfilipou@med.uoa.gr; 4 Second Department of Propaedeutic Surgery, “Laiko” General Hospital of Athens, Medical School, National and Kapodistrian University of Athens, 11527 Athens, Greece; dvmantas@med.uoa.gr

**Keywords:** wound healing, pilonidal excision, secondary intention, translational model, surgical research

## Abstract

**Background/Objectives:** Wound healing is a complex biological process involving coordinated interactions among inflammatory cells, growth factors, extracellular matrix components, and resident tissue cells. Despite significant advances in experimental research, translation of these findings into clinical practice remains limited, partly due to the lack of reproducible and ethically accessible human wound models. Pilonidal disease, a chronic inflammatory condition of the sacrococcygeal region, is frequently treated by surgical excision with healing by secondary intention. The resulting open wound provides a unique opportunity to study the natural progression of human tissue repair. **Methods:** This narrative review examines current knowledge on wound-healing physiology, commonly used experimental wound models, and clinical studies related to pilonidal disease. Evidence from experimental, translational, and clinical literature was evaluated to explore the potential of open pilonidal excision wounds as a standardized human model for wound-healing research. **Results:** Following open excision, healing typically occurs within 4–10 weeks through the classical phases of inflammation, proliferation, and tissue remodeling. During this period, the wound remains externally accessible, allowing repeated clinical observation and serial collection of tissue samples, wound fluid, and exudate. This accessibility facilitates investigation of key biological processes, including angiogenesis, fibroblast proliferation, epithelial migration, cytokine signaling, and extracellular matrix remodeling. Compared with in vitro systems and animal models, the open pilonidal wound offers direct insight into human wound biology under clinically relevant conditions. **Conclusions:** Open pilonidal excision wounds constitute a reproducible and ethically feasible in vivo human model for translational wound-healing research. This model may support biomarker discovery and contribute to the development of new therapeutic strategies for impaired healing and chronic wounds.

## 1. Introduction

Wound healing represents a fundamental biological process that restores the integrity and function of damaged tissues through a tightly regulated interplay of inflammatory, proliferative, and remodeling mechanisms. Although substantial progress has been made in elucidating the molecular and cellular events underlying this process, much of our current understanding stems from experimental models that inadequately replicate human physiology [1]. Traditional in vitro and animal models, while instrumental for mechanistic exploration, lack the anatomical, immunological, and biochemical complexity of human wounds. Consequently, a significant translational gap persists between basic experimental discoveries and their application to clinical wound healing.

Within this context, surgical wounds—especially those healing by secondary intention—offer a unique opportunity for translational investigation. Among these, wounds produced after open pilonidal excision represent an ethically feasible, reproducible, and clinically relevant human model for studying normal tissue repair. Their predictable healing trajectory, accessibility for serial sampling, and controlled clinical environment enable dynamic correlation between biological mechanisms and observable clinical endpoints. This narrative review summarizes the physiological basis of wound healing, evaluates existing non-surgical models, and highlights the potential of open pilonidal excision as a standardized platform for translational human wound-healing research.

## 2. Methods of the Narrative Review

A narrative literature review was conducted to examine the physiological basis of wound healing, existing experimental wound-healing models, and the potential role of open pilonidal excision as a translational human model. Electronic searches were performed in PubMed, Scopus, and Google Scholar from inception until December 2025. The search strategy included combinations of the following keywords: “wound healing”, “wound healing models”, “pilonidal disease”, “pilonidal excision”, “secondary intention healing”, “surgical wound models”, and “human wound healing research.” Both experimental and clinical studies, as well as relevant review articles, were included to provide a comprehensive overview of the topic. Studies not available in English were excluded. Articles were screened based on title and abstract, followed by full-text evaluation when relevant. Priority was given to peer-reviewed studies, well-established experimental models, and clinically relevant investigations. Evidence was interpreted qualitatively, emphasizing biological plausibility, clinical relevance, and translational applicability rather than quantitative synthesis.

## 3. Physiology of Wound Healing

The process of wound healing is a highly orchestrated sequence of events that restores the integrity and function of damaged tissue through four distinct, sequential, yet overlapping stages: (1) hemostasis, (2) inflammation, (3) cellular proliferation, and (4) tissue remodeling. This classification serves as a simplified framework to facilitate understanding and study of a highly dynamic and continuous biological process [2]. In reality, these phases overlap extensively, and it is even possible for different regions within the same wound to be at different stages of healing simultaneously. Successful completion of wound repair requires a complex coordination of cellular and extracellular events, mediated by a network of growth factors, cytokines, and proteolytic enzymes [3]. Major cellular components of the wound healing process are resumed on Table 1.

### 3.1. Hemostasis

Hemostasis represents the first and most immediate phase of wound repair. It begins within seconds of vascular injury and lasts from minutes to hours. Platelets play a central role, not only in halting bleeding but also as key cellular regulators of the entire healing cascade. Upon vascular rupture, platelets aggregate at the site of injury and, through activation of their glycoprotein IIb/IIIa receptors, adhere to the exposed subendothelial collagen to form the initial platelet plug. In parallel, vasoactive substances such as catecholamines and serotonin bind to endothelial receptors, causing local vasoconstriction to minimize blood loss. Simultaneously, smaller capillaries become more permeable, allowing leukocytes, erythrocytes and plasma proteins to extravasate into the wound bed. Activated platelets release a range of bioactive molecules, including cytokines, chemokines, and numerous growth factors such as insulin-like growth factor-1 (IGF-1), transforming growth factor-β (TGF-β), epidermal growth factor (EGF), fibroblast growth factor (FGF), vascular endothelial growth factor (VEGF), and platelet-derived growth factor (PDGF) [4,5].

The polymerization of fibrin strengthens the platelet plug, forming a temporary scaffold that supports the migration of inflammatory and reparative cells—neutrophils, keratinocytes, and fibroblasts—thus setting the stage for the next phase [6].

### 3.2. Inflammation

The inflammatory phase is characterized by the recruitment of neutrophils, macrophages, and lymphocytes into the wound environment [7]. Neutrophils are the first to arrive, engaging in phagocytosis and microbial clearance. They are soon followed by macrophages, which are attracted by degradation products of apoptotic neutrophils. These macrophages, along with lymphocytes, remove cellular debris and orchestrate the immune response [8]. Macrophages typically accumulate within 48 h of injury and persist until the inflammation subsides. Although acute inflammation generally lasts around three days, macrophages continue to exert crucial roles beyond this phase. Their ability to polarize into distinct phenotypes—pro-inflammatory (M1) and anti-inflammatory (M2)—is fundamental for proper healing. M1 macrophages dominate during the inflammatory response, while M2 macrophages promote resolution of inflammation, angiogenesis, and tissue repair [9]. Mast cells also contribute significantly to the regulation of wound healing. These resident immune cells are rapidly activated following tissue injury and release a wide array of mediators, including histamine, proteases, cytokines, and growth factors. Through these mediators, mast cells influence vascular permeability, leukocyte recruitment, and angiogenesis. In addition, mast cells interact with fibroblasts and endothelial cells, thereby contributing later to extracellular matrix remodeling and granulation tissue formation. A delicate equilibrium between pro-inflammatory cytokines (IL-1, IL-6, TNF-α) and anti-inflammatory cytokines (IL-4, IL-10) is essential for successful wound resolution and prevention of excessive tissue damage [10]. Once the wound is cleared of infection and debris, the reparative process can proceed to the proliferative stage.

### 3.3. Proliferation and Tissue Repair

Approximately 48–72 h after injury, the proliferative phase begins, marked by cellular proliferation, extracellular matrix (ECM) formation, angiogenesis, and re-epithelialization. Keratinocytes at the wound edges migrate and proliferate to cover the defect, guided by signals from growth factors and the ECM [11]. These keratinocytes derive from stem cells residing in hair follicle bulbs and apocrine glands. Upon contact with the new matrix, they begin synthesizing a new basement membrane, restoring the epidermal barrier [12]. In wounds healing by secondary intention, the wound bed fills with granulation tissue—a mixture of fibroblasts, endothelial cells, and immature type III collagen. Fibroblasts synthesize provisional ECM components such as procollagen, hyaluronic acid, elastin, and proteoglycans, creating a supportive environment for angiogenesis [13,14]. Endothelial cells re-establish perfusion through capillary sprouting, while keratinocytes continue migrating to seal the surface.

### 3.4. Remodeling and Maturation

This final stage, the remodeling or maturation phase, lasts the longest—often weeks to several months. A central cellular component of the remodeling phase is the myofibroblast, a specialized contractile fibroblast characterized by the expression of α-smooth muscle actin (α-SMA). Myofibroblasts arise from fibroblasts under the influence of transforming growth factor-β (TGF-β) and mechanical tension within the wound microenvironment. These cells contribute to wound contraction and facilitate closure of the defect. In addition, myofibroblasts actively participate in extracellular matrix remodeling through the synthesis and reorganization of collagen fibers. Type III collagen is gradually replaced by type I collagen, which imparts tensile strength to the scar tissue [15]. The collagen matrix becomes more organized and cross-linked, and the wound gains up to 80% of its original tensile strength [16]. Cells that are no longer required undergo apoptosis, the granulation tissue regresses, and the initially hypervascular environment is normalized [17]. The result is the formation of a stable scar and restoration of the skin’s barrier and structural integrity.

## 4. Non-Surgical Wound-Healing Models and Their Comparative Evaluation

Non-surgical wound-healing models encompass a range of experimental systems designed to replicate specific aspects of tissue repair without relying on surgically created wounds. These include in vitro, ex vivo, in vivo animal, and in silico computational approaches, each providing complementary insights into the cellular and molecular mechanisms underlying healing [18].

In vitro models remain foundational tools for exploring the mechanistic basis of wound repair. Common examples include fibroblast and keratinocyte monolayers, scratch assays, and three-dimensional reconstructed skin equivalents that approximate epidermal–dermal interactions. These systems allow precise control of environmental conditions, cytokine exposure, and extracellular matrix composition, making them ideal for studying specific healing phases such as migration, proliferation, and matrix deposition [19]. Their major advantages include reproducibility, cost-effectiveness, and the absence of ethical restrictions. However, they lack essential physiological features such as vascularization, immune cell dynamics, and systemic responses, limiting their translational applicability. Consequently, while in vitro systems are invaluable for hypothesis generation and mechanistic dissection, they cannot fully reproduce the complexity of human wound healing [20].

Bridging the gap between cell culture and living organisms, ex vivo human skin models use full-thickness human skin—typically obtained from surgical discards or cadaveric donors—maintained under organ culture conditions. These models preserve native epidermal and dermal architecture and often retain partial immune competence, enabling the study of processes such as inflammation, re-epithelialization, and extracellular matrix remodeling [21]. Their principal limitation lies in their finite tissue viability and the lack of systemic influences such as angiogenesis and immune recruitment from circulation. Donor variability and logistical constraints also affect reproducibility. Nonetheless, ex vivo models serve as a valuable intermediary between reductionist cell systems and complex in vivo environments.

Although in vivo animal models technically involve the creation of wounds, they are often categorized among non-surgical research systems. This classification reflects the fact that these models employ controlled, small-scale excisions, incisions, or burns under experimental conditions rather than therapeutic surgical procedures. Rodents are the most widely used species owing to their manageable size, low cost, and well-characterized genetics. These models reproduce essential features of the wound-healing cascade within a living organism. Murine systems, in particular, facilitate genetic manipulation, allowing the study of specific signaling pathways. However, significant interspecies differences persist: rodent skin heals largely by contraction through the panniculus carnosus, whereas human wounds rely on granulation and epithelialization [18,22,23]. Larger animals, such as pigs, offer greater anatomical and physiological similarity to humans but entail higher costs and ethical considerations [24,25].

In recent years, computational or in silico models have gained prominence as complementary tools for simulating and predicting wound-healing dynamics. These mathematical frameworks integrate biological data to model processes such as inflammation, cellular migration, and extracellular matrix remodeling [26]. Their advantages include cost-efficiency, the ability to manipulate multiple parameters simultaneously, and freedom from ethical constraints. However, their predictive accuracy depends on the quality and granularity of empirical data, and they often simplify the complexity of biological systems. As such, in silico models are best regarded as hypothesis-generating and hypothesis-testing adjuncts rather than replacements for experimental models [27].

Collectively, these experimental models—ranging from in vitro to in silico—have provided invaluable mechanistic insights into tissue repair (Table 2). Nevertheless, their limitations underscore the need for accessible human models capable of bridging the translational gap between controlled laboratory systems and clinical wound biology [28]. The following sections explore the role of surgical wounds, particularly open pilonidal excisions, in fulfilling this unmet need.

## 5. Surgical Wounds as Research Platform

Although surgical wounds represent a well-defined and clinically relevant context for studying tissue repair, they remain underexplored in wound-healing research. Several factors contribute to this gap. Ethical and practical limitations often restrict invasive sampling or experimental interventions in surgical wounds, as these procedures could interfere with routine patient care or introduce additional morbidity [29]. Furthermore, most surgical wounds heal predictably and within a short period, leaving a narrow observational window and offering limited opportunity to investigate prolonged inflammatory or remodeling phases that are of greater scientific interest.

Another key limitation is the heterogeneity of surgical wounds, which vary widely depending on anatomical site, closure technique, surgical approach, and patient characteristics. This variability complicates standardization of sampling protocols and outcome assessment [30]. In addition, primary closure—commonly used in surgical practice—renders the wound inaccessible once sutured, precluding longitudinal visual or tissue-based monitoring. Regulatory and logistical challenges, including the need for repeated follow-up visits, complex ethical approval processes, and patient compliance issues, further discourage large-scale clinical studies. Finally, research and funding priorities have traditionally focused on chronic wounds such as diabetic, venous, or pressure ulcers, given their significant healthcare and socioeconomic impact, leaving acute surgical wounds comparatively neglected [31].

Given these constraints, the majority of studies investigating normal wound healing have been conducted in vitro, using cultured cell lines [32,33,34], or in animal models such as rabbits, rats, and mice [35,36]. Consequently, human studies on physiological wound healing are scarce. In some cases, postoperative drain fluids from procedures such as colectomies and mastectomies have been used as surrogate samples to study the molecular dynamics of surgical wound healing [37,38,39]. A Finnish research group, for example, investigated human skin re-epithelialization using donor sites of split-thickness skin grafts as standardized wound models, from which serial punch biopsies were obtained [40]. Studies involving pathological conditions—such as pressure ulcers, diabetic foot ulcers, and chronic ulcers of other etiologies—are more common, as these wounds are accessible for repeated sampling and longer-term follow-up [41].

Human wound-healing studies have also been performed to evaluate specific therapeutic interventions, including commercial dressings, topical agents, and negative pressure wound therapy systems [41,42,43]. Moreover, a clinical study examined the effects of platelet-rich plasma (PRP) on the healing of open pilonidal excision wounds, demonstrating the feasibility of using such wounds for translational research [44]. Similarly, Boyce and colleagues utilized open pilonidal excision wounds to investigate the role of lymphocytes in cutaneous repair, further supporting their suitability as an experimental human model [45]. Our research group’s experience from studies on the physiology of wound healing using open pilonidal excisions is very encouraging [46,47].

## 6. Clinical and Surgical Background of Pilonidal Excision

### 6.1. Epidemiology and Surgical Context

Pilonidal disease is a chronic inflammatory and infectious condition of the skin, typically presenting as a cystic lesion within the natal cleft, most often near its upper portion. The estimated incidence is approximately 26 cases per 100,000 individuals, with a mean age of onset of 19 years in females and 21 years in males. The condition demonstrates a marked male predominance, affecting men two to four times more frequently than women [48].

A range of surgical strategies has been developed for its management, including primary closure, various flap-based techniques, and open excision [49]. Contemporary approaches to pilonidal sinus surgery increasingly emphasize minimally invasive or reconstructive techniques that aim to enhance recovery time and patient satisfaction, moving beyond traditional open or simple primary closure methods [50]. Notable advancements include flap procedures such as the Limberg and Karydakis flaps, minimally invasive options like laser ablation and endoscopic-assisted techniques, as well as specialized procedures such as Z-plasty or deroofing designed to modify the natal cleft anatomy [51].

The overarching objective of these approaches is to achieve an optimal balance between durable healing, minimal morbidity, and low recurrence rates. Despite these innovations, open excision with secondary intention healing remains widely practiced, particularly in cases of recurrent or complex disease [52]. Evidence from comparative studies indicates that open techniques may be associated with lower recurrence and infection rates than primary closure methods, whereas minimally invasive approaches, although less morbid, may carry a higher risk of recurrence [53,54]. Ultimately, surgical outcomes depend on multiple factors, including sinus complexity, individual patient anatomy, and the surgeon’s expertise and preferences [55,56]. The open excision technique continues to hold a prominent role in the management of pilonidal disease due to its accessibility, minimal technical demands, low cost, and consistently favorable clinical results [57].

### 6.2. Operative Steps of Open Excision for Pilonidal Disease

During the open excision procedure, the patient is positioned prone or in the jack-knife position, with the buttocks gently separated or taped laterally to provide full exposure of the natal cleft. The operative field is shaved, cleansed, and draped under sterile conditions, and prophylactic antibiotics are administered preoperatively in accordance with institutional protocols [58]. The extent of disease is then delineated by marking the sinus tracts, midline pits, secondary openings, and surrounding skin. In some cases, methylene blue or a similar dye is used to visualize the full trajectory of the sinus system. An elliptical or anatomically contoured incision is subsequently made to encompass the visible sinus openings and affected subcutaneous tissue, extending down to the sacrococcygeal or presacral fascia [59]. The procedure can be well tolerated under local anesthesia [60]. Careful dissection follows, with complete removal of sinus tracts, embedded hairs, granulation tissue, and epithelial remnants, while preserving healthy tissue at the wound margins [61]. Hemostasis is achieved using monopolar electrocautery, diathermy, or argon plasma coagulation as appropriate.

In the open excision technique, the wound is deliberately left unclosed to heal by secondary intention. The cavity is typically packed with sterile gauze or an antiseptic-soaked dressing. Scheduled dressing changes are performed at regular intervals. In some postoperative protocols, sitz baths or antiseptic irrigations are also employed to promote wound cleanliness and comfort [62]. Postoperative care emphasizes meticulous hygiene, continued wound packing, hair removal around the natal cleft, avoidance of prolonged sitting, and regular monitoring for infection or delayed healing.

### 6.3. Standardized Wound Characteristics

Following open excision, a full-thickness defect extending down to the presacral fascia is created and left to heal by secondary intention through granulation. In appropriately selected patients, the wound’s dimensions, depth, and location can be relatively consistent, rendering it suitable for standardized sampling and longitudinal follow-up [63]. The wound bed is typically clean, carries a low risk of contamination, and permits non-invasive observation as well as serial biopsy collection—features rarely achievable in other surgical settings [46,47].

Healing proceeds naturally without surgical approximation of the wound edges, progressing through the well-characterized healing phases, generally completed within 4–10 weeks, depending on wound size and postoperative care [64,65]. In certain cases, however, healing may extend up to three months or remain incomplete, resulting in a chronic non-healing wound. During the reparative process, the wound exhibits sequential morphological changes, including alterations in color, granulation tissue formation, epithelial migration, and contraction. These events are driven by granulation tissue development, angiogenesis, fibroblast proliferation, and collagen matrix deposition.

The predictable clinical trajectory of open pilonidal wounds facilitates direct correlation with biological markers and quantitative assessment of healing progression. Compared with traumatic or chronic wounds, pilonidal wounds following open excision exhibit a relatively uniform healing pattern, enhancing reproducibility and enabling the identification of potential predictive biomarkers for tissue repair outcomes. Unlike wounds managed by primary closure, those healing by secondary intention remain accessible to the external environment, allowing for repeated, minimally invasive evaluation [46,47]. Collectively, these characteristics establish open excision wounds as an ideal in vivo model for investigating tissue repair, immune modulation, and extracellular matrix remodeling.

## 7. Translational Applications

The open pilonidal excision wound offers an ethical and reproducible clinical model for translational studies. Serial sampling of tissue, exudate, and wound fluid allows dynamic assessment of gene expression, proteomic changes, and cellular composition. This approach circumvents the ethical and biological limitations of animal models, providing a direct representation of human wound biology.

Collaboration between surgeons, molecular biologists, and immunologists can turn a routine surgical procedure into a research platform. Using standardized protocols for wound assessment and sample collection, clinical centers can generate biologically meaningful datasets linking molecular signals to healing kinetics [66].

Although pilonidal wounds represent a specific clinical entity, the biological processes governing their repair are largely representative of acute cutaneous wound healing by secondary intention. The sequential activation of inflammatory pathways, fibroblast migration, angiogenesis, extracellular matrix deposition, and epithelialization mirrors the fundamental mechanisms observed in many surgical and traumatic wounds. Insights derived from this model can inform interventions for chronic ulcers, burns, and post-surgical non-healing wounds. Studying how acute pilonidal wounds transition through normal healing may reveal molecular checkpoints that fail in chronic wounds, guiding therapeutic development.

## 8. Ethical and Practical Considerations

As open excision represents a standard surgical treatment for pilonidal disease, the use of this setting as a research model introduces no additional morbidity beyond routine clinical care. Ethical study design necessitates the acquisition of informed consent for biological sampling and adherence to protocols that minimize interference with standard wound management [29]. The collection of samples—such as tissue, wound swabs, or exudate—can be seamlessly incorporated into postoperative dressing changes, requiring minimal additional invasive intervention. This integration enhances the model’s practicality within academic surgical units and facilitates consistent longitudinal follow-up.

Despite its strengths, the model primarily reflects the physiology of acute secondary healing rather than chronic wound pathology. Furthermore, patient-related factors, including age, body mass index, smoking status, and hygiene practices, may introduce biological variability [67,68,69]. Anatomical variations in the natal cleft and differences in wound size may also affect tissue oxygenation, mechanical stress, and bacterial colonization. The relatively homogeneous demographic profile of many patients with pilonidal disease—typically young and otherwise healthy individuals—may reduce the influence of systemic comorbidities that commonly complicate other wound models. Nonetheless, the implementation of standardized protocols and strict inclusion criteria can effectively mitigate these confounding variables, thereby ensuring the reliability and reproducibility of the data obtained.

## 9. Future Directions

Future research should aim to further refine and expand the use of open pilonidal excision as a model for studying human wound healing. Longitudinal biomarker profiling could elucidate the temporal correlation between cellular dynamics, growth factor expression, cytokine activity, and other bioactive molecules with healing duration. The integration of advanced imaging modalities with molecular and histological analyses would enable quantitative assessment of granulation tissue formation, re-epithelialization, and extracellular matrix remodeling [70]. Combining histological assays with immunoblotting techniques could deepen understanding of the cellular and molecular mechanisms underlying tissue repair. Furthermore, the application of artificial intelligence tools holds promise for automating image analysis, identifying predictive patterns, and improving outcome modeling [71]. Comparative studies evaluating healing kinetics and outcomes between open excision and flap closure models, as well as between secondary intention healing and pharmacologically augmented approaches, could provide valuable insights into therapeutic optimization [65,72]. Finally, the creation of standardized biobanks of pilonidal wound tissues and exudates would support multicenter collaborations and foster translational research efforts. Collectively, these initiatives could establish open pilonidal excision as a benchmark human model for wound healing research, facilitating the synthesis and validation of data derived from experimental and preclinical models.

## 10. Conclusions

Open pilonidal excision represents a practical, ethical, and biologically robust model for studying human wound healing in vivo. Unlike conventional experimental systems, this model offers a rare convergence of clinical accessibility, standardized wound morphology, and translational potential. It enables longitudinal sampling and molecular profiling under real-life healing conditions, bridging the divide between preclinical findings and human biology.

By leveraging this model, researchers can investigate the temporal orchestration of cytokines, growth factors, immune mediators, and extracellular matrix dynamics that underpin successful healing. Furthermore, integrating advanced imaging, omics technologies, and artificial intelligence-driven analytics may transform open pilonidal excision into a cornerstone for translational wound-healing research. Through multidisciplinary collaboration and standardized methodologies, this approach has the potential to redefine how normal and pathological wound healing are studied, ultimately guiding the development of targeted therapeutic strategies for chronic and non-healing wounds.

## Figures and Tables

**Table 1:** Major cellular components of wound healing.

Cell Type	Role in Wound Healing	Key Mediators
Platelets	Initiate hemostasis and release Growth Factors	PDGF, TGF-β, VEGF
Neutrophils	Early inflammatory response, microbial clearance	ROS, proteases
Macrophages	Phagocytosis, cytokine signaling, tissue repair regulation	IL-1, TNF-α, TGF-β
Mast cells	Modulate inflammation, angiogenesis, fibroblast activation	Histamine, tryptase, cytokines
Fibroblasts	Extracellular matrix synthesis, granulation tissue formation	Collagen III, fibronectin
Myofibroblasts	Wound contraction, matrix remodeling	α- SMA, collagen I
Endothelial cells	Angiogenesis	VEGF
Keratinocytes	Re-epithelialization	EGF, KGF

**Table 2:** Comparative Evaluation of Wound Healing Research Models.

Model Type	Advantages	Limitations	Translational Value
In Vitro Models	Controlled conditions, reproducible, low cost	Lack vascularization, immune interactions, systemic factors	Low
Ex Vivo Human Skin	Preserves native skin architecture	Limited viability, no systemic immune responses	Moderate
Animal Models	Whole-organism physiology, genetic manipulation possible	Species differences in healing mechanisms	Moderate
Computational Models	Predictive simulations	Dependent on Empirical Data	Variable
Surgical Wounds	Human physiology	Ethical, practical, heterogeneity	Moderate—High
Open Pilonidal Excision	Human physiology, accessible wound, serial sampling	Patient variability, Limited to acute healing	High

## Data Availability

Data is contained within the article. Further inquiries can be directed to the corresponding author(s).
